# Preparation of Fe–N co-doped carbon-based catalysts and their influence on tetracycline degradation properties†

**DOI:** 10.1039/d5ra00189g

**Published:** 2025-03-07

**Authors:** Yang Tao, Tiyang Xiao, Qing Fu, Bin Miao, Sanying Hou, Guowen Peng, Yiyang Xiong, Manzhen Tang

**Affiliations:** a School of Resource Environment and Safety Engineering, University of South China Hengyang 421001 China 852376775@qq.com; b School of Chemistry and Chemical Engineering, University of South China Hengyang 421001 China tysgying@163.com; c School of Mechanical and Aerospace Engineering, Nanyang Technological University Singapore 639798 Singapore

## Abstract

A high-performance Fe–N co-doped carbon-based catalyst was prepared to activate PMS by a one-step pyrolysis using ferbam, melamine and sucrose. The best catalyst Fe–N/C exhibited high catalytic activity against PMS at an ultra-low catalyst dosage (50 mg L^−1^), and the TC decomposition rate reached 92.3% within 30 min. The results demonstrated that graphite N, pyridine N, Fe^0^ and Fe_3_C were the main active substances for catalyzing PMS. Free radical quenching analysis and EPR experiments further proved that the O_2_˙^−^ served as the main active oxygen species. Finally, the environmental hazard of the intermediate product was studied by ECOSAR system.

## Abstract 2

A high-performance Fe–N co-doped carbon-based catalyst was prepared to activate PMS degrading tetracycline by a one-step pyrolysis using ferbam, melamine and sucrose.

## Graphical abstract

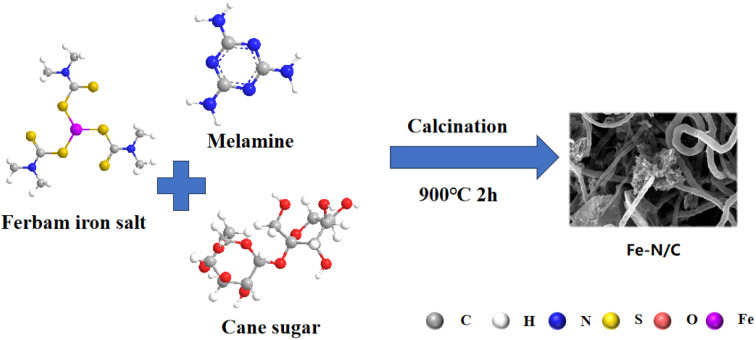

## 1. Introduction

At present, antibiotics have been widely used not only in the prevention and treatment of bacterial infectious diseases in the medical field, but also used as growth inhibitors in agriculture, animal husbandry and aquaculture.^1,2^ Antibiotics can induce bacterial resistance and contribute to its spread, posing harm to both the ecological environment and human health. At the same time, it also affects the structure and function of microbial population in the water body, thereby harming the growth and development of organisms in the water body, and causing damage to the human urinary tract, kidney and other organs.^3^ Therefore, the efficient degradation of antibiotic wastewater has become an important process.

Due to its non-selective degradation of organic pollutants, the advanced oxidation process (AOP) based on peroxymonosulfate (PMS) has attracted more and more attention in water treatment research. Compared with the traditional Fenton process, SO_4_˙^−^ produced by persulfate activation owned a longer half-life (30–40 μs),^4^ higher oxidation potential (*E*_0_ = 2.5–3.1 V), broader pH (2.0–8.0) and better oxidation selectivity.^5^ However, PMS itself exhibited insufficient oxidative capacity against organic pollutants, and some methods such as ultrasonic,^6,7^ ultraviolet,^8,9^ carbon substrate derived catalysts,^10,11^ transition metal ion-guided catalysts (Co^2+^, Fe^2+^, or Ni^2+^)^12^ and their cooperative derivative catalysts have been employed in activating PMS.^13^ Doping transition metals and nitrogen in carbon-based materials has been considered to be an effective strategy for preparing catalysts that could activate PMS. These catalysts were commonly denoted as M–N–C catalysts.^14^ Nitrogen doping could enhance the surface alkalinity of the carbon material and increase the electrochemical activity. This, in turn, strengthened the adsorption of PMS and improved the efficiency of electron transfer from the catalysts to the contaminants. The addition of transition metals further regulated the electronic structure, improved the catalytic performance, and promoted the recycling of the catalyst.^15^ Among them, iron-based catalysts are widely used for the modification of carbon-based catalysts due to their wide availability and low cost. It had been shown that the iron doping (such as single-atom, cluster, particle) could significantly improve the low reaction rate caused by the deficiency of active sites and poor structural stability in carbon-based catalysts.^16–20^ For instance, the Fe–CN-650 prepared by Zeng *et al.*^21^*via* Fe^3+^ and 1,10-phenanthroline ligand could remove 100% of SMX in 20 min with excellent cycling performance. Compared with the FeN_4_ ligand, FeN_3_O_1_ showed better interaction with PMS. However, metal–ligand based catalyst-loaded catalysts suffer from the disadvantage of high temperature sintering, which reduces the dispersion and exposure of active iron sites. It is worth noting that metal particles possess a more stable structure, especially Fe_3_C. Xiao *et al.*^22^ Nitrogen-doped nanotube catalysts encapsulating Fe/Fe_3_C particles synthesized by supramolecular self-assembly possessed superior degradation performance for TC with reduced iron loss.

However, the Fe–N/C catalysts reported so far still encounters some constraints. Their preparation procedures are complex, leading to high cost and insufficient synergistic catalytic activity. The issue arises from the large and aggregational Fe-containing particles, which hinder effective interaction between nanoparticles and active nitrogen species.^23–25^ Therefore, it is urgent to adopt efficient and facile preparation strategies to rationally design three-dimensional porous Fe–N/C catalysts with well distributed Fe-nanoparticles for PMS-activation.

Hence, in this study, a high-performance Fe–N co-doped carbon-based catalyst (Fe–N/C) with iron source (ferbam) was prepared by one-step pyrolysis strategy for activating PMS to the tetracycline hydrochloride (TC) degradation. The elemental structure and variations of the catalysts were investigated thoroughly and the catalytic sites were explored in conjunction with a series of degradation experiments, including temperature, catalyst dosage, contamination concentration, PMS dosage and common interfering factors in natural water environment. The types of free radicals were corroborated by radical trapping experiments and electron paramagnetic resonance spectroscopy, and the main degradation mechanisms were derived. Finally, possible routes in TC degradation were investigated and the toxicity of intermediates was analyzed.

## 2. Experimental

### 2.1. Synthesis of catalysts

**Fig. 1:**
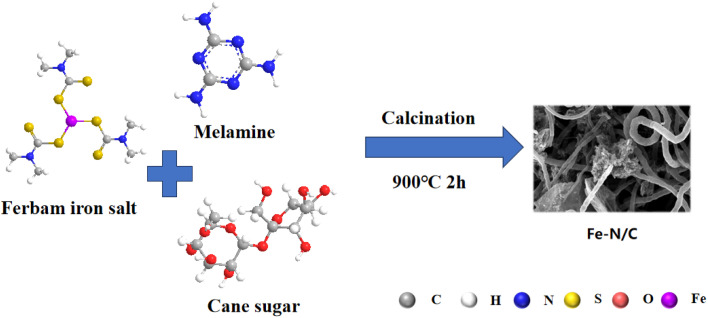
Synthesis process of the Fe–N/C catalysts.

The Fe–N/C catalysts were prepared using a simple grinding and calcination process as shown in Fig. 1. Typically, 0.11 g ferbam, 2 g melamine and 0.3 g sucrose were added to the mortar and grind to dry thoroughly. The dried samples were placed in a quartz boat and with N_2_ at a heating rate of 5 °C min^−1^ to 900 °C for 2 h, which was represented as Fe–N/C. Under the same conditions, N/C and C were prepared with the same method but without the addition of iron or nitrogen sources. In addition, by varying the calcination temperature to 800 and 1000 °C and the amount of melamine of 1and 3 g, some comparison samples were prepared, which denoted as Fe–N/C-800, Fe–N/C-1000, Fe–N_1_/C and Fe–N_3_/C.

### 2.2. Catalytic experiment

Typically, the reaction temperature was 25 °C. In a 100 ml oxidation system, the obtained Fe–N/C catalysts were added to a certain concentration of tetracycline solution and dispersed by sonication. After the addition of PMS, the timed extraction of the sample was started. The samples were filtered by a 0.45 μm polytetrafluoroethylene (PTFE) filter and the absorbance was determined by UV-1900i with a wavelength of 357 nm. Quenchers including *tert*-butanol (TBA), furfuryl alcohol (FFA), methanol (MeOH), and *p*-benzoquinone (PBQ) were used to verify the oxidation mechanism. All experimental tests were repeated three times to minimize possible errors.

### 2.3. Assessment standards

Calculation formula of TC degradation rate:1
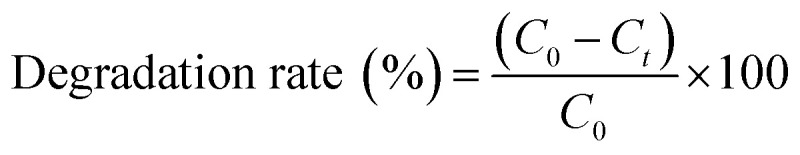


*C*_0_ represents the initial concentration of tetracycline and *C*_*t*_ is the concentration of tetracycline remaining at time *t*.

## 3. Results and discussion

### 3.1. Characterization of catalysts

The morphology of the catalysts (Fe–N/C, N/C and C) was characterized by scanning electron microscopy (SEM). Fig. S1a and b† depicted that a graphene-like structure was formed after the N doping, and the carbon nanotubes (CNTs) were further occurred after Fe/N co-doping. The element mapping results in Fig. S1(d–g)† and EDS spectra (Fig. S1h†) confirmed that C, O, N and Fe elements were successfully doped and evenly distributed in the Fe–N/C catalyst.

**Fig. 2:**
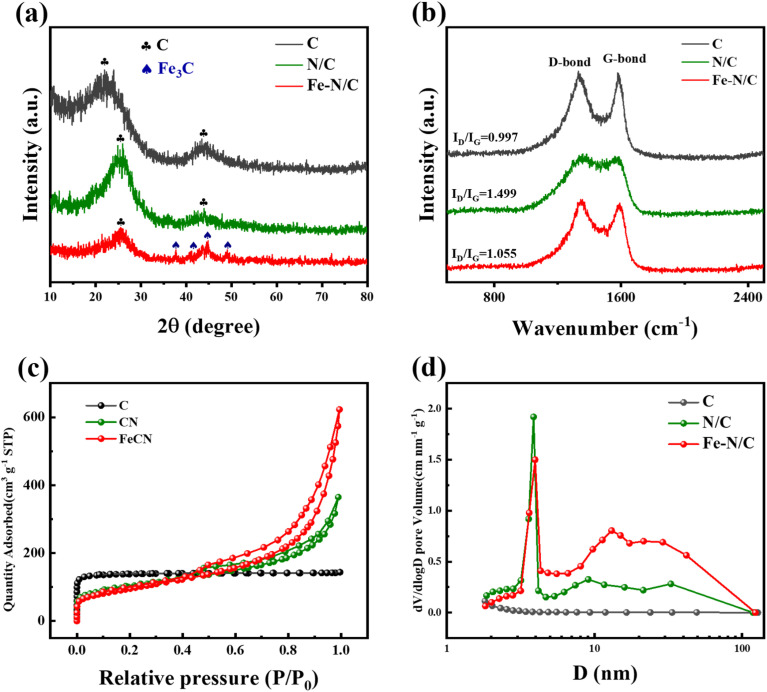
(a) XRD patterns, (b) Raman spectra, (c) nitrogen adsorption–desorption curves (d) pore size distributions of catalysts.

The material structure and crystal composition of the different catalysts were measured by X-ray diffraction (XRD) (Fig. 2a). The C and N/C catalysts exhibited two similar diffraction peaks at 26.5 and 44°, which corresponded to the (002) and (101) planes of graphitic carbon, respectively.^26^ For the Fe–N/C catalyst, diffraction peaks were occurred in the range of 37.83, 40.15, 43.38 and 44.94°, which represented the Fe_3_C phase, illustrating the successful doping of Fe elements.^27^

The degree of defects in the catalysts was investigated by Raman spectroscopy (Fig. 2b). All catalysts showed two characteristic peaks near 1355 cm^−1^ and 1575 cm^−1^, which corresponded to the positions of defective carbon (D-band) and graphitic carbon (G-band), respectively. The ratio of *I*_D_/*I*_G_ was commonly used to assess the degree of defects in the carbon structure.^28^ The *I*_D_/*I*_G_ of Fe–N/C, N/C and C catalysts were 1.055, 1.499 and 0.997, respectively. The Fe–N/C exhibited the lower *I*_D_/*I*_G_ value than N/C due to the loading of iron on the surface of carbon substrate, which could destroy the graphene-like structure of the carbon material partially. C catalyst had the lowest *I*_D_/*I*_G_ value, suggesting that the degree of disorder in the carbon material might increase due to the embedding of N and Fe and lead to the creation of additional defect sites in the carbon network.^29,30^

The N_2_ adsorption–desorption curves of C, N/C and Fe–N/C were given in Fig. 2c. The C catalyst showed a typical type I isotherm, indicating a rich microporous structure was formed in C materials,^31^ and a typical type IV curve was occurred in Fe–N/C and N/C catalyst, respectively, illustrating the mesoporous characteristics of the catalysts. Table S1† illustrated that the total pore volume of N/C (0.5631 cm^3^ g^−1^) was increased compared to C, which might be related to the melamine as a blowing agent to produce abundant pore structures through gas bubbling. Meanwhile, the Fe–N/C possessed the highest porosity and average pore size. However, C showed an extremely poor pore distribution compared to Fe–N/C and N/C (Fig. 2d). In addition, Fe–N/C exhibited smaller specific surface area (341.2839 m^2^ g^−1^) compared to N/C (361.9232 m^2^ g^−1^). It was shown that iron doping resulted in a reduction in the specific surface area of the catalyst due to clogging of the pores.^32^

**Fig. 3:**
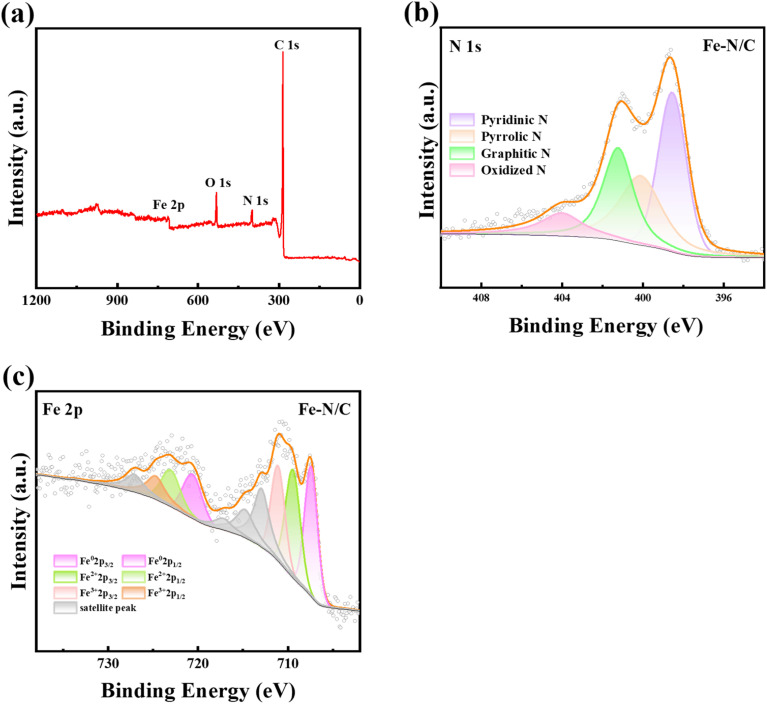
(a) Elemental composition, (b) N 1s XPS diagram and (c) Fe 2p XPS diagram of the Fe–N/C.

The elemental composition of the Fe–N/C was discussed by X-ray photoelectron spectrum (XPS). Fig. 3a showed that S 2p (168.46 eV), C 1s (285.36 eV), N 1s (400.35 eV), O 1s (532.24 eV), and Fe 2p (710.17 eV) were existed in Fe–N/C. However, the S content was so low (Table S2†), which could be ignored, corresponding with the SEM results (Fig. S1h†). As shown in Fig. 3b, four peaks located at 398.55, 400.11, 401.23 and 404.02 eV, which represented pyridine N, pyrrole N, graphite N and oxidized N, respectively,^33^ with corresponding contents of 34.04, 28.92, 26.6 and 10.43%, respectively. Graphite N was more resistant to high temperatures and activated PMS by promoting electron transfer to produce reactive species. At the same time, pyridine N also promoted π-electron transfer on its adjacent sp^2^ carbon, generating more free radicals.^34^ Fe 2p spectra (Fig. 3c) showed six peaks of 707.49, 710.89, 715.29, 720.179, 723.89 and 728.69 eV, which was regarded as Fe^0^ (2p_3/2_), Fe^2+^ (2p_3/2_), Fe^3+^ (2p_3/2_), Fe^0^ (2p_1/2_), Fe^2+^ (2p_1/2_) and Fe^3+^ (2p_1/2_), with contents of 17.06, 16.20, 13.39, 8.53, 8.10 and 6.69%, respectively.

### 3.2. Catalytic activity of catalysts

#### 3.2.1 Degradation properties of catalytic systems

The Fe–N/C catalysts with different carbonization temperatures (800, 900 and 1000 °C) were employed to activate PMS towards the oxidation of tetracycline. As shown in Fig. S2(a),† As the catalyst annealing temperature increased from 800 to 900 °C, the TC removal efficiency increased from 89.4% to 92.3%. When the pyrolysis temperature was further raised to 1000 °C, the catalyst performance was similar with that carbonizing at 900 °C. The Fe–N_1_/C (with 1 g melamine addition), Fe–N/C (with 2 g melamine addition), and Fe–N_3_/C (with 3 g melamine addition) with different melamine ratio were further tested for their adsorption and degradation performance. As depicted in Fig. S2(b),† the TC removal efficiency was increased as the melamine ratio increased, which was similar with previous reports,^35,36^ suggesting the higher nitrogen content could increase the active sites of the catalyst and promoted the activation of PMS.

**Fig. 4:**
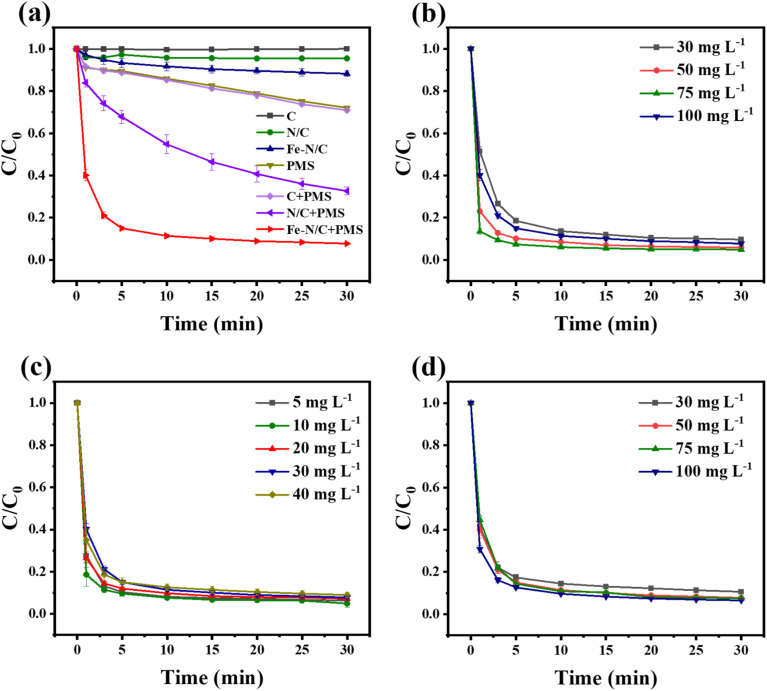
(a) Removal efficiency for TC in different systems, and (b) catalyst dosage effect, (c) TC concentration effect and (d) PMS dosage effect in Fe–N/C/PMS system.

The adsorption degradation experiments of C, N/C and Fe–N/C were conducted. As shown in Fig. 4a, the adsorption efficiency of C, N/C and Fe–N/C were 0.01, 4.5, and 10%, respectively, which indicated that the adsorption capability of catalysts were very weak. For PMS alone, the TC removal efficiency was 30% within 30 min, suggesting that the degradation of TC by PMS alone was limited, which could be caused by the self-decomposition of PMS. When N element was doped, the degradation effect reaches 65% in 30 min. This might be related to the high electronegativity of N atom, which could change the charge distribution of C atom. In addition, the catalytic effect was the best when Fe and N were doped at the same time, and the removal effect of TC reached 92.3% within 30 min, which attributed to the Fe and N co-doping altered chemical inertness of the carbon substrate. Furthermore, the performance of Fe–N/C for TC degradation was compared with other different catalytic materials that have been reported in previous literature. As illustrated in Table S3,† home-made Fe–N/C displayed excellent catalytic performance of PMS with TC removal rate of 92.3% in 30 min at ultra-low catalyst dosage (50 mg L^−1^) and high TC concentration (30 mg L^−1^) condition.

#### 3.2.2 Interference experiments in Fe–N/C system

Fig. 4b showed that the degradation efficiency of TC improved from 90% to 95% with catalyst concentration increased from 0.03 g L^−1^ to 1.00 g L^−1^, which implied that more catalyst could enhance the activation of PMS and accelerate the oxidation reaction of electron transfer and free radical generation. The removal effect of initial TC concentration to the system was investigated. As shown in Fig. 4c, when the TC dosage was 5 mg L^−1^, the degradation efficiency of TC reached up to 93.6%, and still stable at 92.3% as TC concentration increased to 30 mg L^−1^. However, when the TC concentration was further raised to 40 mg L^−1^, the removal rate of TC slightly reduced to 90.1%, indicating that our catalyst is suitable for a wide range of TC concentrations. The dosage of PMS could also greatly affect the removal efficiency of tetracycline. As depicted in Fig. 4d, the removal rate of TC improved from 89% to 93.7% with the dosage of PMS increased from 0.25 g L^−1^ to 1 g L^−1^, this might be due to the extra production of reactive oxygen species in the system, which accelerated the TC removal process. In addition, the decomposition of PMS contributed more oxidizing groups.

**Fig. 5:**
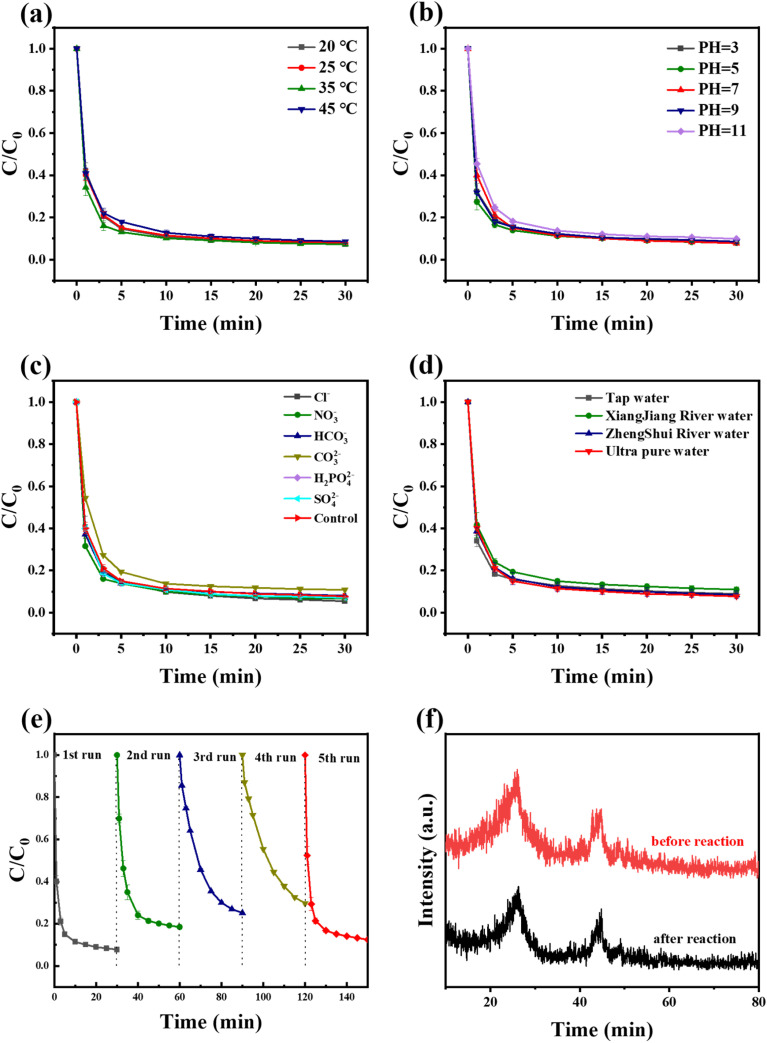
Effect factors for Fe–N/C/PMS: (a) temperature, (b) pH, (c) inorganic ions, (d) different water bodies and (e) five cycles experiment of Fe–NC (f) XRD changes of the used Fe–N/C.

The influence of temperature on the TC removal properties in Fe–N/C/PMS systems was further investigated in Fig. 5a. The decomposition efficiency of TC had a small influence when the temperature of the system increased from 25 °C to 45 °C, indicating that the catalytic ability of the system was not affected by temperature. Considering the above results, the experiments in the next part of the study would be carried out under the optimal condition of 0.5 g per L PMS, 0.05 g per L Fe–N/C, 30 mg per L TC and at 25 °C.

In Fe–N/C/PMS system, the initial pH of the system was a prominent factor for the degradation performance which was shown in Fig. 5b. In a wide range of pH (3–11), all of the degradation rate of TC achieved above 89%. However, when the pH of the solution was 11, the removal efficiency of TC was most affected. This might be attributed to the excessive OH^−^, which make the surface of the catalyst negatively charged. Additionally, the decomposition of PMS under alkaline conditions was also a factor. This decomposition was not conducive to the reaction inside the system.^37^

The presence of inorganic anions in the water environment might react with the active species generated by PMS activation and influenced the degradation efficiency of organic pollutants. The effects of Cl^−^, NO_3_^−^, HCO_3_^−^, CO_3_^2−^, H_2_PO_4_^2−^, SO_4_^2−^ on the TC decomposition were investigated. It could be found in Fig. 5c that the TC decomposition was promoted when Cl^−^ was introduced into the reaction system, and the removal efficiency of TC reached 94.5%. The higher the concentration of Cl^−^, the more active chlorine free radicals (˙Cl, Cl_2_, HO–Cl) generated,^38^ which improved the decomposition of TC. In addition, the hydrolyzed solution of CO_3_^2−^ was alkaline and exhibited an inhibitory effect on the reaction, which was in keeping with the results of pH interference experiments. However, other ions (SO_4_^2−^, NO_3_^−^, *etc.*) had no significant influence on the decomposition of TC, suggesting that the catalyst system had practical adaptability.

The effects of water bodies to the Fe–N/C/PMS system were investigated in Fig. 5d. The Fe–N/C catalysts exhibited stable TC removal ability in Zhengshui River water, tap water and ultrapure water. Although the removal efficiency of TC in Xiangjiang water was inhibited to some extent, it still remained above 85%, which reflected that the system had a great anti-interference performance to the actual water body.

The influence of different pollutants (methylene blue (MB), levofloxacin (LVX)) on the degradation profiles of Fe–N/C/PMS system was explored. As shown in Fig. S3.† The results showed that 80.6% of LVX and 100% of MB could be removed by the Fe–N/C/PMS system within 30 min. This illustrates that the Fe–N/C catalyst were more effective in the degradation of MB.The catalytic difference might be caused by the differences in the physicochemical properties and substituent groups of the organic pollutants.

#### 3.2.3 Stability assessment of Fe–N/C/PMS system

The stability of catalyst was studied by multiple cycle oxidation process. As shown in the Fig. 5e, the TC decomposition rate within 30 min decreased from 92.3% to 70.3% after four consecutive runs, and the degradation rate of TC recovered to 87.5% after Fe–N/C calcined at 550 °C under Ar for 2 h, which indicated that the catalyst displayed the ability of activation and regeneration, and it could be heated and activated to improve the catalytic effect after several times of use. As seen in Fig. 5f, the XRD of the used catalyst exhibited no change, confirming that the catalyst possessed high structural stability. The Fe leaching was tested by inductively coupled plasma-optical emission spectrometer (ICP-OES). Fig. S4† showed the leaching amount of iron ions during the five cycles. Among them, the leaching amount of iron ions decreased sequentially in the first four cycles, with a maximum of 0.719 mg L^−1^, while the fifth cycle showed a slight decrease of 0.65 mg L^−1^, which was related to the removal of adsorbents on the surface of the catalyst in the high-temperature state. However, as a whole, the iron ion leaching rate of the five cycles was less than 0.8 mg L^−1^, which was much lower than the 10 mg L^−1^ of the Chinese total iron emission standard (GB/T31962-2015).

#### 3.2.4 Analysis of active species

**Fig. 6:**
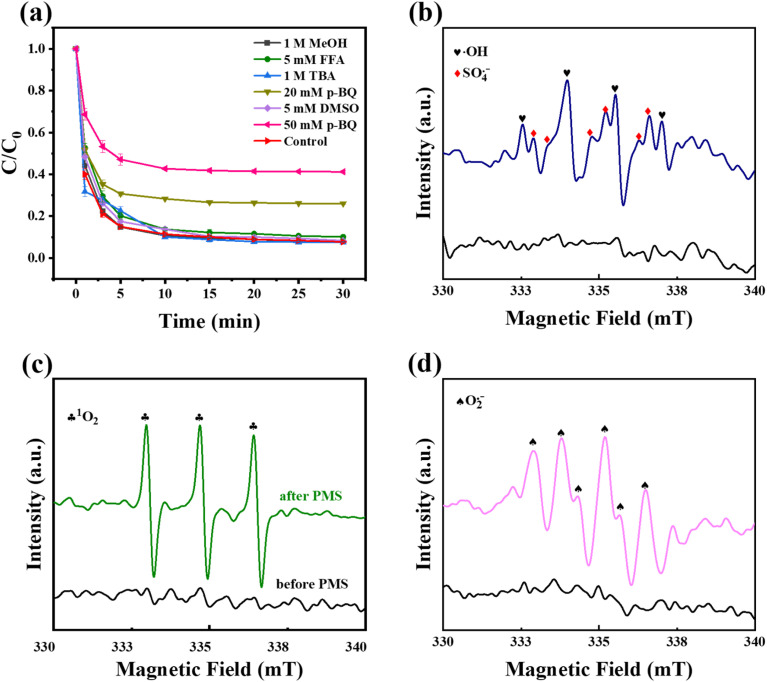
(a) Effect of selective scavengers on TC removal and the EPR spectra of (b) DMPO–˙OH and DMPO–SO_4_˙^−^, (c) TEMP–^1^O_2_, (d) DMPO–O_2_˙^−^.

In order to identify the active substances in Fe–NC/PMS system, quenching experiments were carried out. MeOH can capture SO_4_˙^−^ and OH^−^and TBA is used to selectively uptake ˙OH. FFA can determine singlet oxygen (^1^O_2_) and *p*-BQ is selective for O_2_˙^−^. It could be seen from Fig. 6a that the catalytic degradation rate of TC showed no significantly change after the addition of 1000 mM TBA and MeOH, indicating that the degradation effect of ˙OH and SO_4_˙^−^ generation on TC was negligible. After adding 5 mM FFA, the removal efficiency of TC reduced from 92.3% to 88.9%, indicating that ^1^O_2_ might be present in the reaction system. With the adding of 20 mM *p*-BQ, it could be found that the remove efficiency of TC decreased to 74.3%. While the dosage of *p*-BQ was further improved to 50 mM, the removal of TC was just 40%, suggesting that the O_2_˙^−^ radical based pathway was the dominant pathway.

The presence of different reactive oxygen species in the Fe–N/C/PMS system was verified by electron paramagnetic resonance experiments (EPR). ˙OH, SO_4_˙^−^ and O_2_˙^−^ were captured by DMPO as spin trapping agent. TEMP was used as a spin catcher to detect ^1^O_2_. As shown in Fig. 6b, adding the DMPO to a blank water system with catalysts exhibited no EPR signal. On the contrary, when the Fe–N/C and PMS was present, weak signals belonging to DMPO–SO_4_˙^−^ and DMPO–˙OH were detected. It could be seen that the stronger signal, with strength of 1 : 2 : 2 : 1, corresponded to ˙OH, while the weaker signal around ˙OH was attributed to SO_4_˙^−^. This suggests that Fe–N/C/PMS promoted the presentation of SO_4_˙^−^ and ˙OH during the activation of PMS. On the other hand, in Fig. 6c, three characteristic peaks of TEMP–^1^O_2_ were detected, which proved the existence of ^1^O_2_. Fig. 6d showed that six characteristic signal peaks were detected in the reaction system, suggesting the presence of O_2_˙^−^. Hence, it could be concluded that both the ^1^O_2_ and free radical pathways were involved in the degradation of TC in the system. Especially, in the free radical pathway, O_2_˙^−^ contributed a major role in the oxidative degradation of TC compared with ˙OH and SO_4_˙^−^, which attributed to that oxygen in the air got electrons to form O_2_˙^−^, activating PMS to degrade TC.

Recent studies have also shown that the electron transfer from the target pollutant to the oxidant in carbon-based materials can accelerate the decomposition of the target pollutant.^39,40^ In order to investigate whether the electron transfer exists in the Fe–NC system, CV curves of catalysts and open circuit voltage (OCP) were explored to investigate the electrochemical performance of the catalysts. As shown in Fig. S5(a),† Fe–N/C possessed the largest inner circle area, indicating that it exhibited the largest electrochemical active surface area (ECSA).^22^ Furthermore, open circuit voltage test (OCP) of the system in Fig. S5(b)† showed that the initial potential of Fe–N/C was about 0.41 V, which increased to about 0.98 V after the addition of PMS, and then decreased significantly after the addition of TC, suggesting that the intermediate (Fe–N/C/PMS*, which has a higher oxidation potential) triggered the electron transfer path.

#### 3.2.5 Main mechanism analysis

**Fig. 7:**
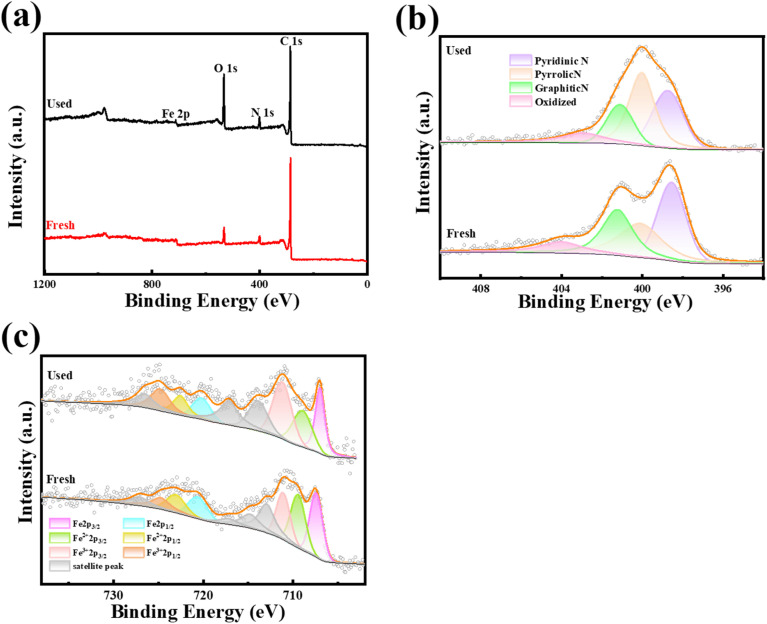
XPS diagram of the before and after reaction of Fe–N/C: (a) full spectrum diagram, (b) N 1s and (c) Fe 2p.

In order to investigate the important role played by the catalyst to the activation of PMS, the elemental changes in the catalyst before and after its use were compared in Tables S4 and S5.† It was showed that a decrease in graphite N (from 26.59% to 18.37%) and pyridine N (from 34% to 30%) and an increase in pyrrole N (from 28.9% to 40.3%), illustrating that graphite N and pyridine N might be the main active sites involved in PMS activation. Among them, graphite N and Fe_3_C could improve the electrical conductivity of the carbon substrate and promote the self-decomposition ability of PMS. In addition, some studies suggested that the Lewis base site produced by pyridine N reduced the energy barrier of adjacent carbon atoms, accelerated electron transfer, and could promote the production of ˙OH and SO_4_˙^−^.^41^ As shown in Fig. 7c and Table S5,† Fe^0^ and Fe^2+^ decreased by 4.77 and 4.8%, respectively. The content changes of Fe^3+^ and Fe^2+^ illustrated that these iron species were also involved in the reaction. The reduction of Fe^0^ attributed to the reaction with HSO_5_˙^−^ to produce Fe^2+^ (eqn(2)), and Fe^0^ could be slowly oxidized to Fe^2+^ (eqn (3)). Meanwhile, Fe^2+^ could be further oxidized by HSO_5_˙^−^ to Fe^3+^, SO_4_˙^−^ and ˙OH (eqn (4) and (5)), and it was worth noting that O_2_ promoted the conversion of Fe^2+^ to Fe^3+^ to produce O_2_˙^−^ (eqn (6)). At the same time, Fe^3+^ could be reduced to form Fe^2+^ (eqn (7)), and the cycle of Fe^2+^/Fe^3+^ on the reaction process promoted the decomposition of TC.

**Fig. 8:**
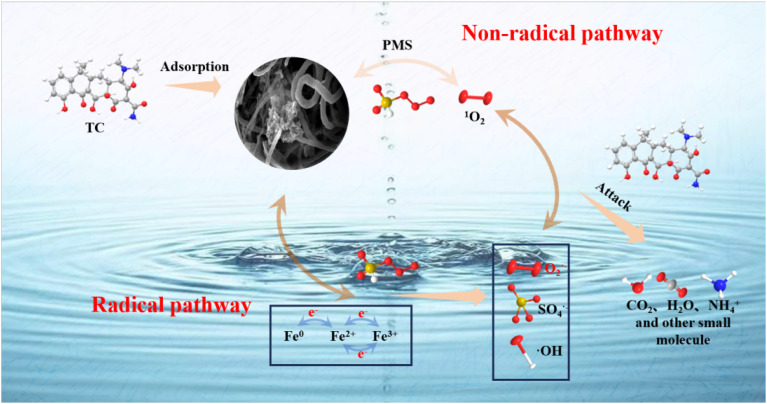
TC decomposition mechanism in the Fe–N/C/PMS system.

The possible TC decomposition mechanism in the Fe–N/C/PMS system was depicted in Fig. 8. At all, Fe–N/C/PMS exhibited excellent catalytic properties, mainly benefiting from the graphite N, pyridine N and Fe species (Fe^0^ and Fe_3_C). In addition, hydrolysis of PMS could produce free radicals. These active sites facilitated the activation of PMS, generating strong oxidative groups that degraded and mineralized TC into small molecules and water.

2 Fe^0^ + 2HSO_5_^−^ → Fe^2+^ + 2OH^−^ + 2SO_4_˙^−^

3 Fe^0^ → Fe^2+^ + 2e^−^

4 Fe^2+^ + HSO_5_^−^ → Fe^3+^ + SO_4_˙^−^ + OH^−^

5 OH^−^ + SO_4_˙^−^ → ˙OH + SO_4_˙^2−^

6 Fe^2+^ + O_2_ → Fe^3+^ + O_2_˙^−^

7 2Fe^3+^ + Fe^0^ → 3Fe^2+^

#### 3.2.6 Degradation intermediate and toxicity analysis of TC

**Fig. 9:**
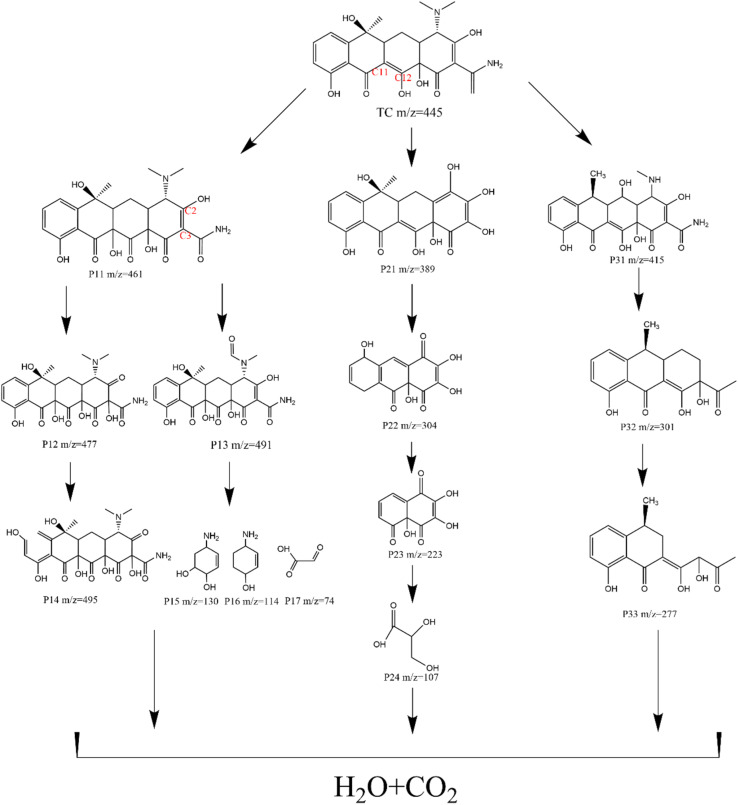
Possible degradation pathway of TC in the Fe–N/C/PMS system.

The mineralization of TC under the Fe–N/C/PMS treatment was investigated by the determinations of TOC. The results indicated that the TOC removal achieved 25.2% (Table S6†). To further investigate the TC degradation mechanism, the intermediates produced during the TC degradation in the coupling system was explored by the Liquid Chromatography Mass Spectrometry (LC-MS). Fourteen main degradation by-products were described in Fig. S6.† Three possible TC degradation paths were suggested, as shown in Fig. 9. In the first pathway, the intermediate P11 had a *m*/*z* of 461, resulting from the initial 1,3-dipole cycloaddition to the C11–C12 double bond and rearrangement with ˙OH at the C12 position.^42^ Then, the C2–C3 double bond of P11 was attacked by ˙OH to form the intermediate P12.^43,44^ P14 were formed by hydroxyl groups associated with the degradation of aromatic ring A. The intermediate product P13 (*m*/*z* = 491) was produced by the continuous attack product P11 of ˙OH.^45^ As the reaction progresses, due to a series of ring-opening reactions and the loss of functional groups, the resulting intermediates were further degraded into small molecules of organic matter (*m*/*z* = 130, *m*/*z* = 114, *m*/*z* = 74, *etc.*), and finally completely decomposed into CO_2_ and H_2_O. In the second pathway, demethylation of nitrogen methyl in TC was accompanied by the removal of the C–N bond, resulting in P21 (*m*/*z* = 389). Subsequently, P21 lost its methyl group and opened the ring, giving P22 (*m*/*z* = 304). Smaller intermediates were then cyclized step by step, including P23 (*m*/*z* = 223) and P24 (*m*/*z* = 107). In the third pathway, TC dropped an OH group under free radical attack to form P31 (*m*/*z* 415), and then the free radical further oxidized the electron rich conjugated double bonds to form P32 (*m*/*z* = 301).^46^ The ring-opening cleavage of material ions resulted in the formation of products P33 (*m*/*z* = 277).^47^ Finally, the small molecules and some intermediates were further decomposed into CO_2_ and H_2_O.

The acute and chronic toxicity of the intermediates was calculated by ECOSAR software as shown in Table S7.† According to the GHS Toxicological Classification System, the concentration of LC_50_/EC_50_/ChV represented their degree of hazard.^48^ Its concentration below 1 mg L^−1^ is red (very toxic), between 1 and 10 mg L^−1^ is orange (toxic), between 10 and 100 mg L^−1^ is blue (harmful) and greater than100 mg L^−1^ is green (harmless). For acute toxicity, most intermediates and TC were little toxic. In terms of chronic toxicity, daphnia showed greater toxicity than TC, such as P12, P14, P32. Overall, most of the intermediates had much lower toxicity and might eventually be degraded to non-toxic water or CO_2_, suggesting that the system possessed some environmental value.

## 4. Conclusion

In this study, Fe–N/C catalyst was successfully prepared by one simple calcination strategy for activating PMS to degrade tetracycline. The optimal Fe–N/C displayed an improved catalytic performance, which can be ascribed to the accelerating effect of reactive species (graphite N, pyridine N, Fe^0^ and Fe_3_C) on the PMS activation. In addition, the Fe–N/C also exhibited good catalytic performance in natural water, illustrating the widespread prospects practical application of Fe–N/C. The ROS burst experiments and EPR showed that the Fe–NC/PMS system produced ˙OH, SO_4_˙^−^, O_2_˙^−^, ^1^O_2_ and electronic transfer and the main ROS were O_2_˙^−^. Three possible degradation pathways were explored by LC-MS, and the acute and chronic toxicity of TC and its degradation intermediates was analyzed using ECOSAR software. The process proposed offers a carbon-based catalyst with high catalytic activity, which will be expected to develop new ideas for the application of M–N–C catalyst in PMS activation.

## Data availability

The data supporting this article have been included as part of the ESI.†

## Conflicts of interest

There are no conflicts to declare.

## Supplementary Material

RA-015-D5RA00189G-s001
