# Supplementary material for: Preparation of Fe–N co-doped carbon-based catalysts and their influence on tetracycline degradation properties
Source: RSC Adv. 2025 Mar 7;15(10):7307–17. doi: 10.1039/d5ra00189g (PMC11886776; doi:10.1039/d5ra00189g)
Supplement: RA-015-D5RA00189G-s001 [file RA-015-D5RA00189G-s001.pdf]

Supplementary Information (SI) for RSC Advances.
This journal is © The Royal Society of Chemistry 2025

Preparation of Fe-N co-doped carbon-based catalysts and their influence on tetracycline degradation properties

Yang Tao[a#], Tiyang Xiao[b#], Fu qing[b], Bin Miao[c], Sanying Hou[b*], Guowen Peng[a*], Yiyang Xiong[b], Manzhen Tang [b]

[a]School of Resource Environment and Safety Engineering, University of South China, Hengyang 421001, China.

[b]School of Chemistry and Chemical Engineering, University of South China，Hengyang 421001, China.

[c]School of Mechanical and Aerospace Engineering, Nanyang Technological University, Singapore 639798

Materials

Ferbam ($C_9H_{18}FeN_3S_6$ (99.99 %), melamine ($C_3N_6H_6$, 99 %), peroxonosulfate ($KHSO_5 \cdot 0.5KHSO_4 \cdot 0.5K2SO_4$, AR), sucrose, tert-butanol (TBA, AR), tetracycline (TC, AR), p-benzoquinone (p-BQ, 99 %), methanol (MeOH, AR), $Na_2H_2PO_4$ (AR), L-histidine (L-His, AR), NaCl (AR) are provided by Shanghai Aladdin Chemical Reagent Co, LTD, China. Na_2CO_3(AR), Na_2SO_4(AR), $NaHCO_3$ (AR) were supplied by Macklin Biotechnology Co, LTD (Shanghai). $NaNO_3$(AR) and KI(AR) are provided by China Pharmaceutical Group Chemical Reagents Co, LTD. The ultra-pure water used in the

* Corresponding author: Sanying Hou Email: tysgying@163.com
* Corresponding author: Guowen Peng Email: 852376775@qq.com
# Authors contributed equally.

experiment came from the ULUPURE water Unit (Chengdu, Sichuan, China) Instrument.

Characterization

The samples were characterized using following techniques: TEM (TalosF200XG2), X-ray photoelectron spectroscopy (Thermo Scientific K-Alpha+), Nitrogen adsorption-desorption isotherm measurement (ASAP2020+2.00), Raman spectrometer (WITec alpha300R, Germany), X-ray diffraction (TD 3500, Dandong), and Liquid chromatography-mass spectrometry (UPLC H-C1ASS, Waters).

Electrochemical performance testing

Open-circuit voltage tests were carried out in a three-electrode system. The catalyst and 0.25 % nafion-isopropanol mixed slurry were first prepared. A glassy carbon electrode coated with 20 ul of catalyst slurry was used as the working electrode, Ag/AgCl and carbon rods were the reference and counter electrodes, respectively, and the reaction was carried out in deionized water.

Other electrochemical properties were measured in 0.1 M Na_2SO_4. Linear sweep voltammetry curves (LSV) were tested at -1-1 V, impedance (EIS) was determined in the frequency range of 0.01-106 Hz, and cyclic voltammetry curves (CV) were tested in the range of -0.7-0.2 V.

Table S1 Physical structural properties of C, N/C, and Fe-N/C

Sample	BET Surface Area (m^2 g^{-1})	Micropore Volume (ml g^{-1})	Tota pore volume of pores (mL g^{-1})	D_{BJH}(nm)
C	554.2561	0.2115	0.2214	2.5368
N/C	361.9232	0.0899	0.5631	7.1933
Fe-N/C	341.2839	0.0138	0.9015	10.0206

Table S2 Content of the element of Fe-N/C

Sample	C(at.%)	N(at.%)	O(at.%)	Fe(at.%)	S(at.%)
Fe-N/C	85.47	8.04	5.4	0.83	0.26

Table S3 Comparison of TC degradation by different catalysts/PMS systems

Materials	Dosage	Degradation efficiency	Reference
SA Fe-N-C	[Catalyst]=100 mg L^{-1} [TC]=20 mg L^{-1} [PMS]=0.15 mM	91 % in 60 min	1
SA Fe-g-C_3N_4(600)	[Catalyst]=100 mg L^{-1} [TC]=10 mg L^{-1} [PMS]=0.5 mM	93.29 % in 40 min	2
Fe/Fe_3C@NCNT-800	[Catalyst]=50 mg L^{-1} [TC]=30 mg L^{-1} [PMS]=500 mg L^{-1}	95.2 % in 30 min	3
Fe/Fe_3C@NCNF-800	[Catalyst]=30 mg L^{-1} [TC]=20 mg L^{-1}	90.8 % in 30 min	4

	[PMS]=1 mM		
Fe/ N-CS-0.1/PMS	[Catalyst]=100 mg L^{-1} [TC]=20 mg L^{-1} [PMS]=0.5 mM	92.7 % in 15 min	5
0.1Fe/N-C	[Catalyst]=70 mg L^{-1} [TC]=20 mg L^{-1} [PMS]=130 mg L^{-1}	88.9 % in 30 min	6
Fe-N/BC	[Catalyst]=50 mg L^{-1} [TC]=30 mg L^{-1} [PMS]=500 mg L^{-1}	91.3 % in 60 min	7
Fe-N-CS-800	[Catalyst]=200 mg L^{-1} [TC]=20 mg L^{-1} [PMS]=1 mM	93.74 % in 12 min	8
FSBC800	[Catalyst]=100 mg L^{-1} [TC]=10 mg L^{-1} [PMS]=50 mg L^{-1}	90.9 % in 30 min	9
Fe-N/C	[Catalyst]=50 mg L^{-1} [TC]=30 mg L^{-1} [PMS]=500 mg L^{-1}	92.3 % in 30 min	This work

Table S4 Change of N 1s before and after the reaction

Sample	N 1s			
	Pyridinic N	Oxidized N	Graphitic N	Pyrrolic N
Fresh	34 %	10.51 %	26.59 %	28.9 %
Used	30 %	11.33 %	18.37 %	40.3 %

Table S5 Change of Fe 2p before and after the rection

Sample	Fe_{2p}						
	Fe^0 $2p_{3/2}$	Fe^{2+} $2p_{3/2}$	Fe^{3+} $2p_{3/2}$	Satellite	Fe^0 $2p_{1/2}$	Fe^{2+} $2p_{1/2}$	Fe^{3+} $2p_{1/2}$
Fresh	17.06 %	16.2 %	13.39 %	30.03 %	8.53 %	8.1 %	6.69 %
Used	13.87 %	12.57 %	19.26 %	31.7 %	6.04 %	6.93 %	9.63 %

Table S6 Change of TOC before and after the reaction

System	TC removal (%)	TOC removal (%)
Only PMS (30 min)	30	7.3
PMS+Catalyst (30 min)	92.3	25.2

Table S7 Toxicity of TC and its degradation intermediates

Compound	Acute Toxicity (mg/L)			Chronic Toxicity (mg/L)		
	Fish (LC_{50})	Daphnid (LC_{50})	Green Algae (EC_{50})	Fish	Daphnid	Green Algae
TC	1.31E+4	1.06E+3	1.89E+3	2.49E+3	59.9	474

P11	1.30E+3	126	157	142	8.48	44.9
P12	56.7	7.02	5.38	2.95	0.597	1.83
P13	1.15E+5	5.07E+4	1.32E+4	8.33E+3	2.44E+3	1.96E+3
P14	28.7	3.76	2.58	1.27	0.337	0.917
P15	3.41E+3	278	485	629	15.8	123
P16	539	50.2	67.5	66.6	3.24	18.7
P17	190	240	96.7	147	1.41	22.2
P21	3.18E+4	1.47E+4	4.68E+3	2.44E+3	809	775
P22	181	104	81.7	18	10.5	22
P23	922	487	269	82.8	38.8	60
P24	1.56E+5	7.01E+4	2.01E+4	1.16E+4	3.59E+3	3.14E+3
P31	1.85E+4	1.45E+3	2.73E+3	3.82E+3	79.7	671
P32	13.5	8.05	17.5	1.44	0.996	3.07
P33	23.9	11.8	30.5	2.44	1.36	4.83

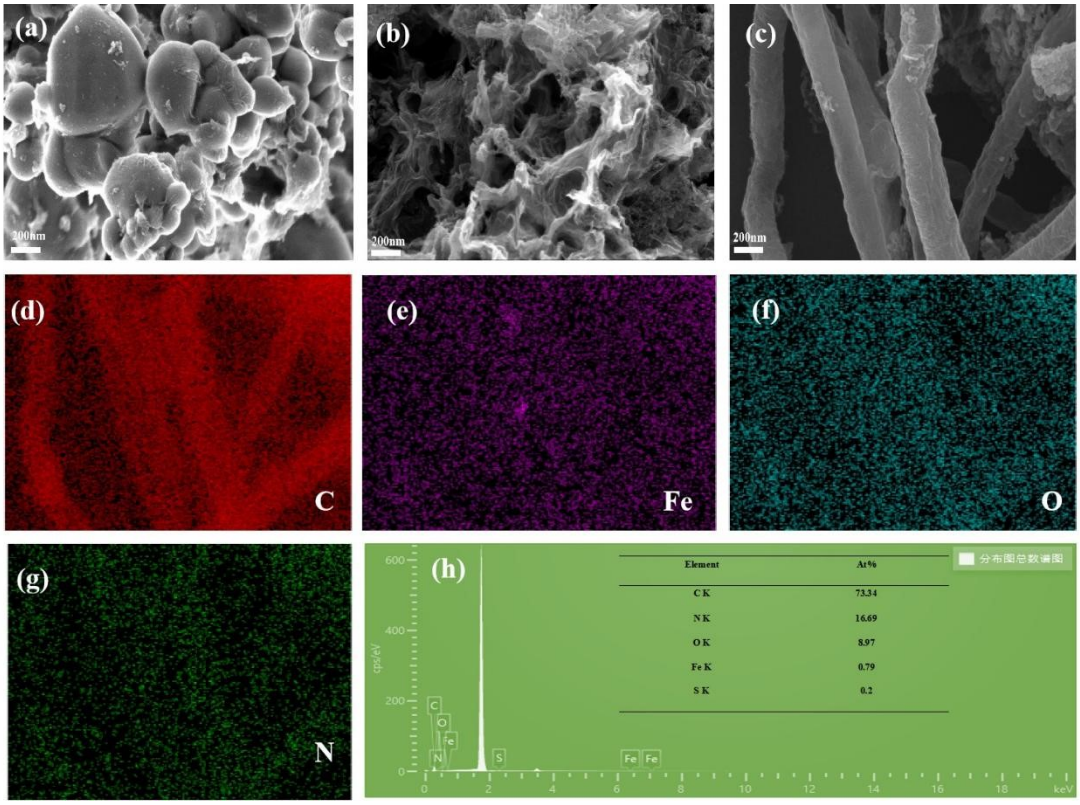

Fig. S1 The SEM of C (a), N/C (b), Fe-N/C (c) at 200 nm, the element distribution of Fe-N/C(d-g) and EDS spectrum of Fe-N/C

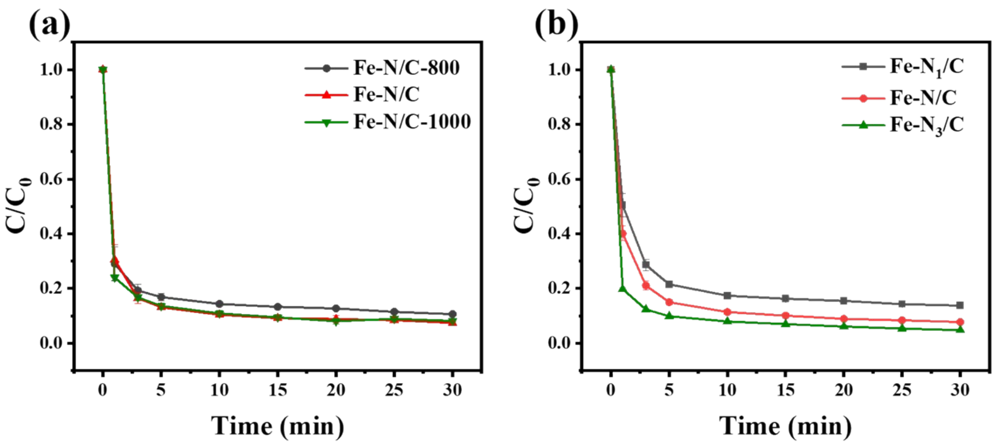

Fig. S2 TC removal curves at (a) different temperatures and (b) melamine doping

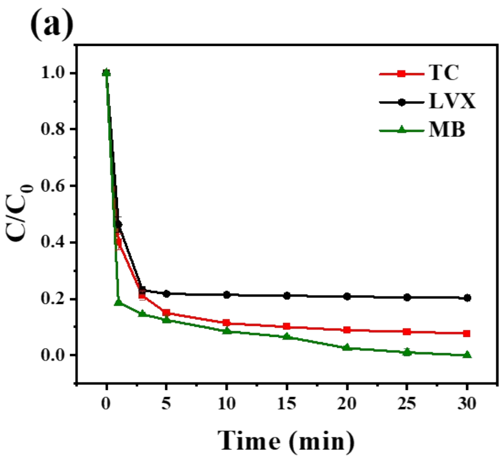

Fig. S3 Degradation curves of the Fe-N/C+PMS system for different pollutants

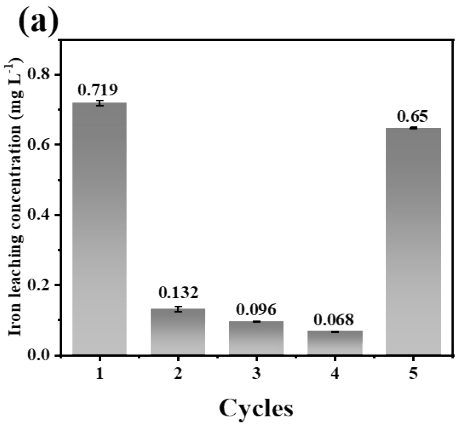

Fig. S4 Leaching concentration of ferrous ions after cyclic testing

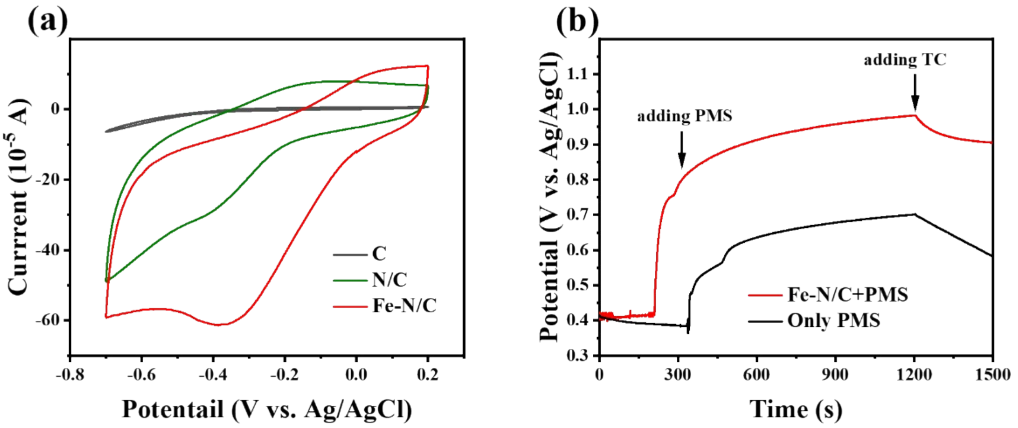

Fig. S5 Cyclic voltammetry curves (a) and open-circuit voltage testing (b) of different materials

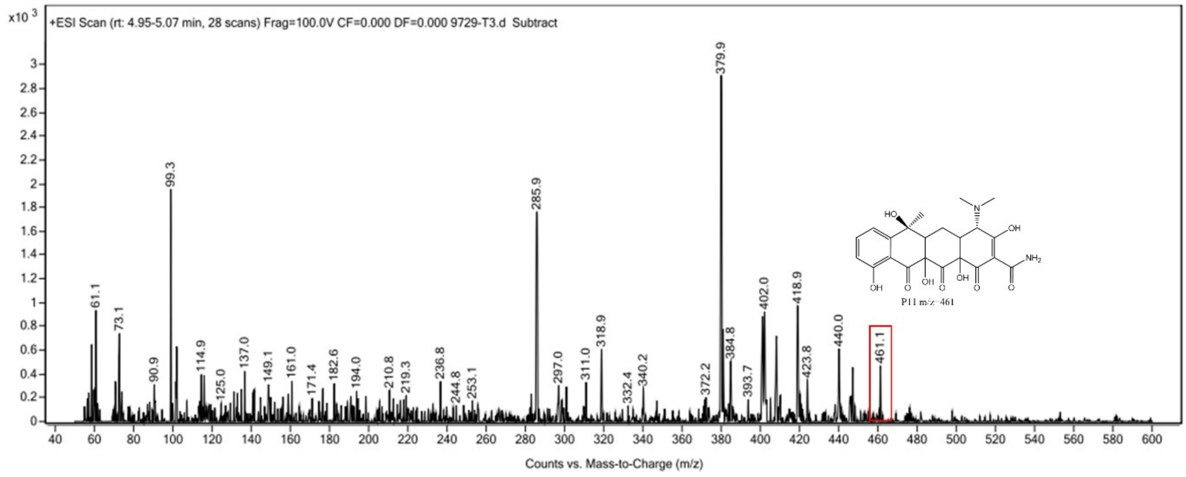
x10 3
+ESI Scan (rt: 4.95-5.07 min, 28 scans) Frag=100.0V CF=0.000 DF=0.000 9729-T3.d Subtract
61.1 73.1 90.9 99.3 114.9 125.0 137.0 149.1 161.0 171.4 182.6 194.0 210.8 219.3 236.8 244.8 253.1 285.9 297.0 311.0 318.9 332.4 340.2 372.2 379.9 384.8 393.7 402.0 418.9 423.8 440.0 461.1
P11 m/z=461
Counts vs. Mass-to-Charge (m/z)

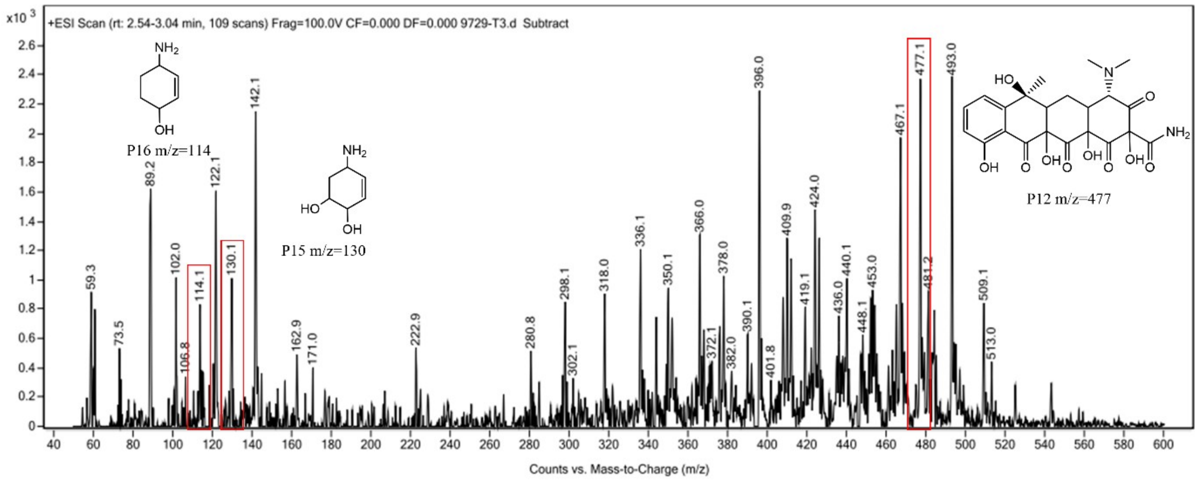
x10 3
+ESI Scan (rt: 2.54-3.04 min, 109 scans) Frag=100.0V CF=0.000 DF=0.000 9729-T3.d Subtract
P16 m/z=114
P15 m/z=130
P12 m/z=477
59.3 73.5 89.2 102.0 106.8 114.1 122.1 130.1 142.1 162.9 171.0 222.9 280.8 298.1 302.1 318.0 336.1 350.1 366.0 372.1 378.0 382.0 390.1 396.0 401.8 409.9 419.1 424.0 436.0 440.1 448.1 453.0 467.1 477.1 481.2 493.0 509.1 513.0
Counts vs. Mass-to-Charge (m/z)

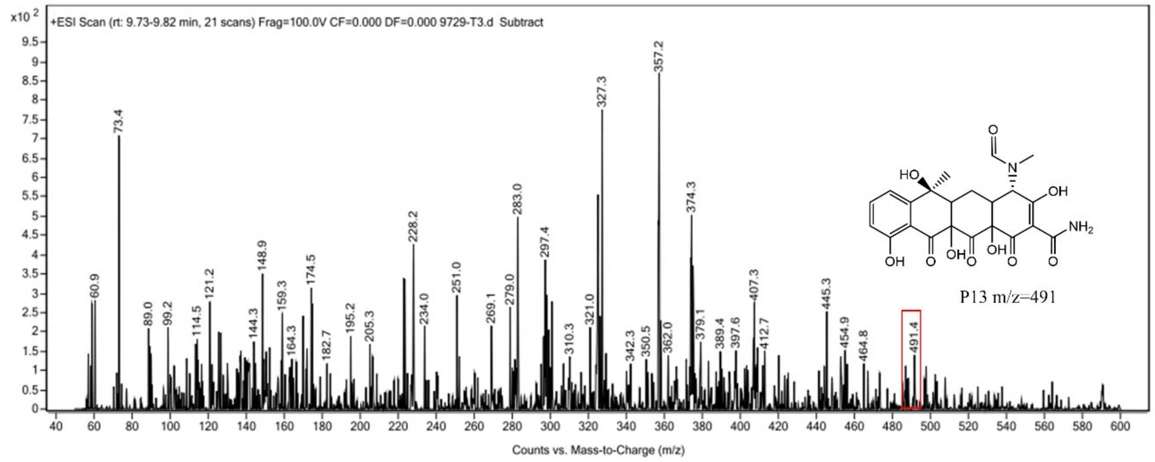
+ESI Scan (rt: 9.73-9.82 min, 21 scans) Frag=100.0V CF=0.000 DF=0.000 9729-T3.d Subtract
x10 2
Counts vs. Mass-to-Charge (m/z)
P13 m/z=491

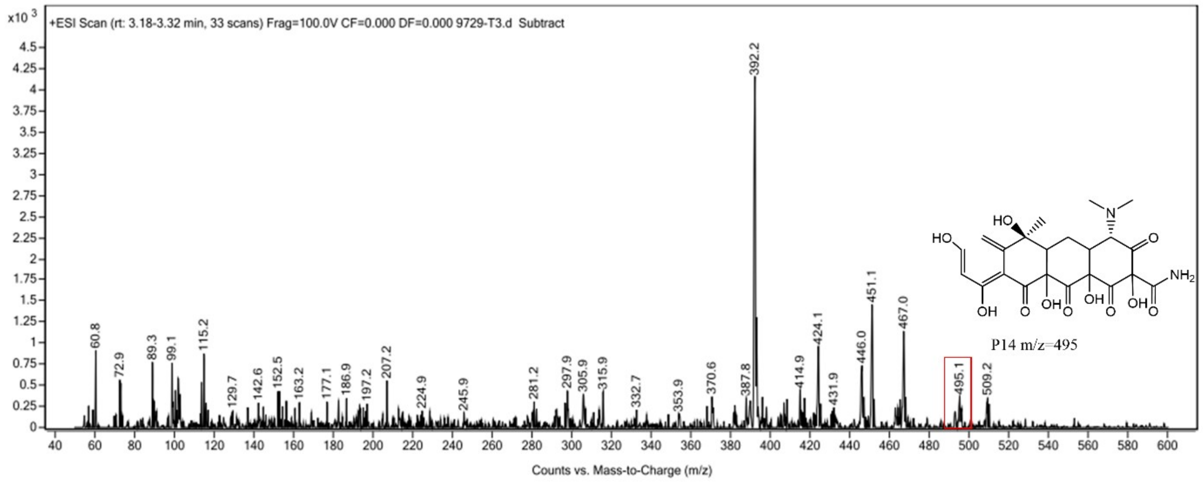
+ESI Scan (rt: 3.18-3.32 min, 33 scans) Frag=100.0V CF=0.000 DF=0.000 9729-T3.d Subtract
x10 3
Counts vs. Mass-to-Charge (m/z)
P14 m/z=495

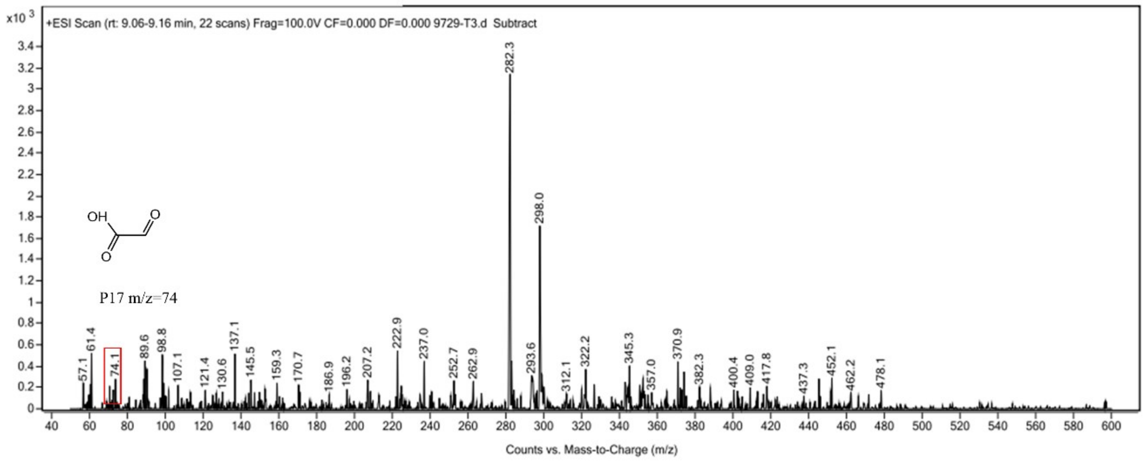
x10 3
+ESI Scan (rt: 9.06-9.16 min, 22 scans) Frag=100.0V CF=0.000 DF=0.000 9729-T3.d Subtract
P17 m/z=74
Counts vs. Mass-to-Charge (m/z)

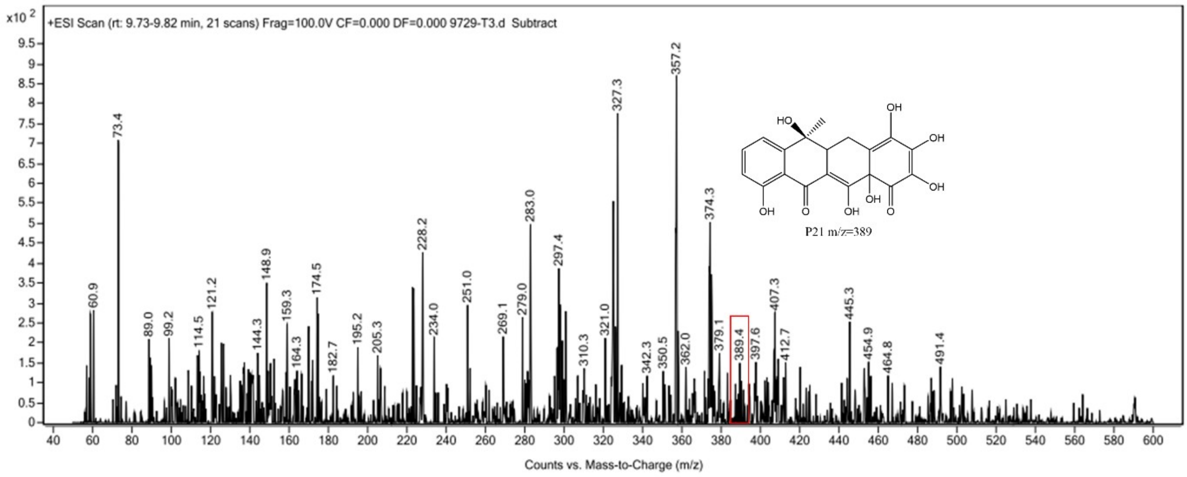
x10 2
+ESI Scan (rt: 9.73-9.82 min, 21 scans) Frag=100.0V CF=0.000 DF=0.000 9729-T3.d Subtract
P21 m/z=389
Counts vs. Mass-to-Charge (m/z)

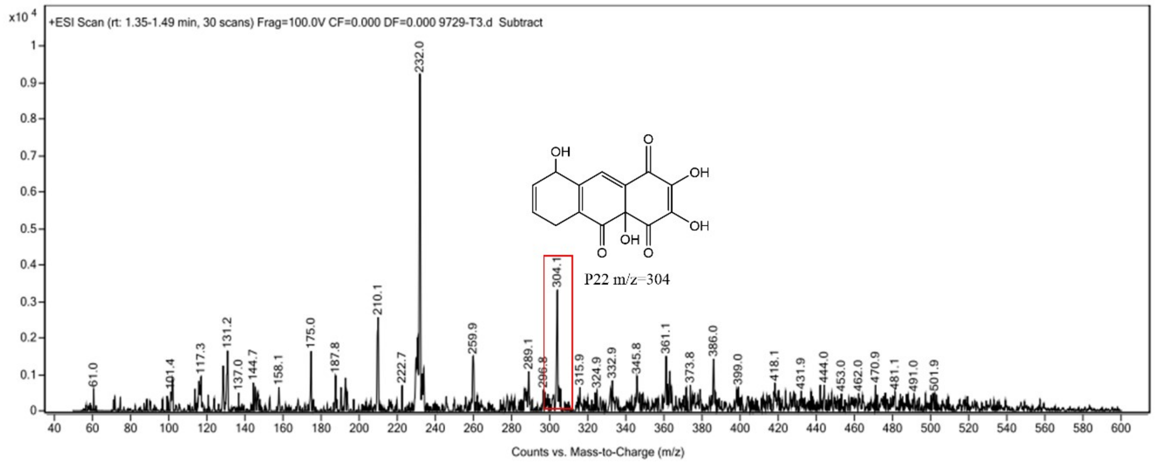
x10 4
+ESI Scan (rt: 1.35-1.49 min, 30 scans) Frag=100.0V CF=0.000 DF=0.000 9729-T3.d Subtract
232.0
210.1
304.1
259.9
175.0
131.2
386.0
361.1
P22 m/z=304
Counts vs. Mass-to-Charge (m/z)

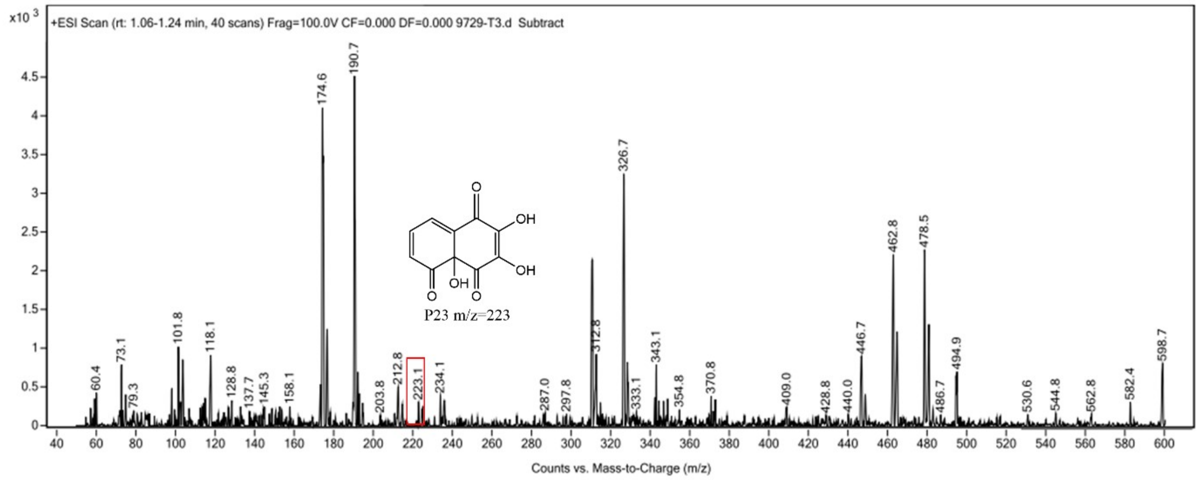
x10 3
+ESI Scan (rt: 1.06-1.24 min, 40 scans) Frag=100.0V CF=0.000 DF=0.000 9729-T3.d Subtract
190.7
174.6
326.7
462.8
478.5
223.1
P23 m/z=223
Counts vs. Mass-to-Charge (m/z)

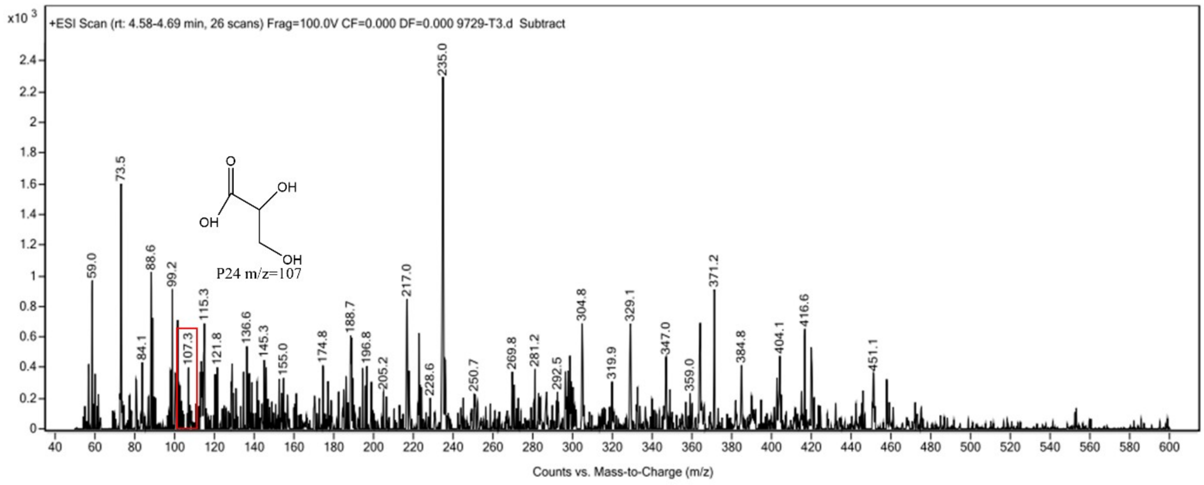

+ESI Scan (rt: 4.58-4.69 min, 26 scans) Frag=100.0V CF=0.000 DF=0.000 9729-T3.d Subtract
x10 3
P24 m/z=107
Counts vs. Mass-to-Charge (m/z)

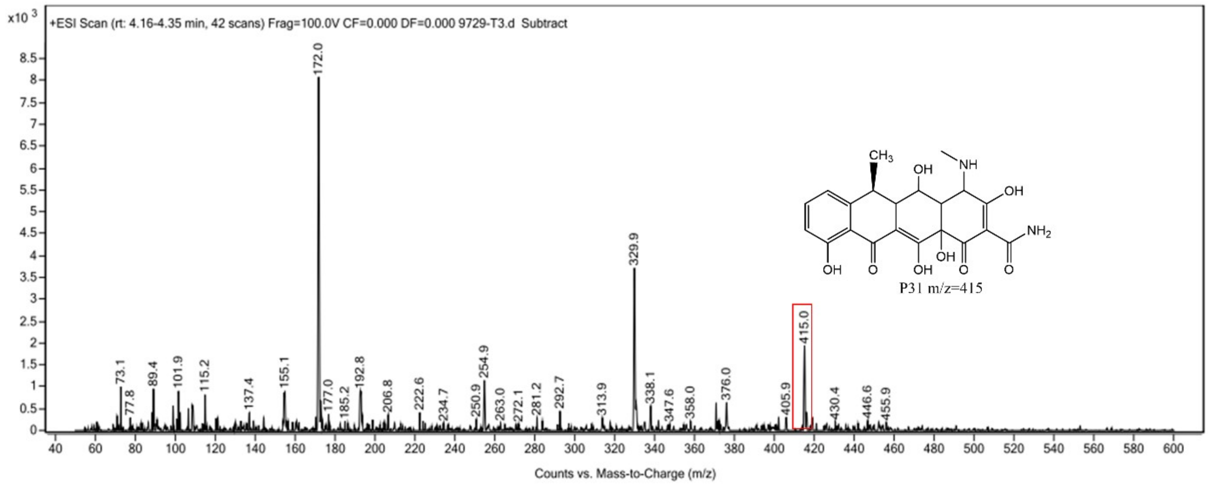

+ESI Scan (rt: 4.16-4.35 min, 42 scans) Frag=100.0V CF=0.000 DF=0.000 9729-T3.d Subtract
x10 3
P31 m/z=415
Counts vs. Mass-to-Charge (m/z)

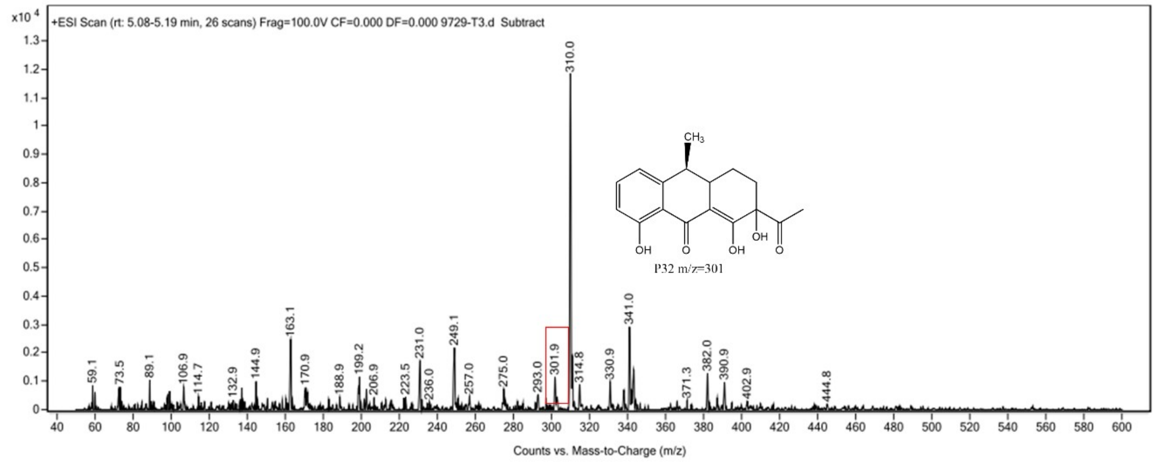

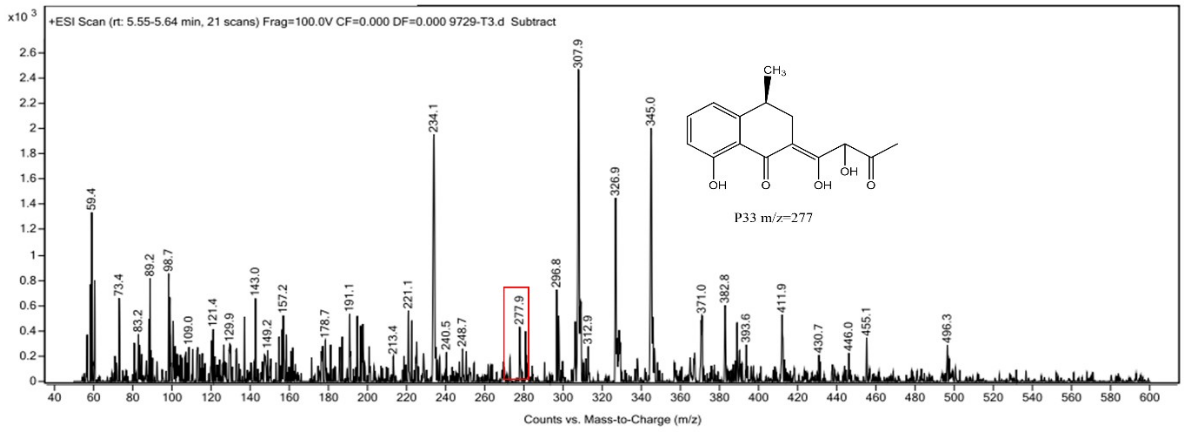

Fig. S6 Mass spectra of degradation intermediates.

References

1 S. Fang, Y. He, X. Cao, Y. Li, L. Gu, W. Mao, B. Wang and H. Zhang, *J. Environ. Chem. Eng*, 2024, **12**, 112584.

2 X. Peng, J. Wu, Z. Zhao, X. Wang, H. Dai, L. Xu, G. Xu, Y. Jian and F. Hu, *Chem. Eng. J*, 2022, **427**, 130803.

3 T. Xiao, Y. Tao, S. Hou, H. Wang, J.-Q. Xie, Y. Chang, Q. Fu, K. Du and S. Zhou, *Sep. Purif. Technol*, 2025, **356**, 129916.

4 K. Zhu, W. Xia, D. He, J. Huang, H. He, L. Lei, W. Chen and X. Liu, *J. Colloid Interface Sci*, 2022, **609**, 86–101.

5 Z. Yan, W. Gao, C. Zhong, Q. Jiao, X. Zhao and J. Liu, *Sep. Purif. Technol*, 2024, **351**, 128075.

6 Z. Liu, A. Kang, Z. Du, H. Chen and X. Yao, *J. Environ. Chem. Eng*, 2023, **11**, 111576.

7 S. Hou, H. Hu, Q. Fu, T. Xiao, J.-Q. Xie, S.-H. Chan, M. He, B. Miao and L. Zhang, *Sep. Purif. Technol*, 2024, **333**, 125980.

8 W. Chen, J. Huang, Y. Shen, K. Zhu, L. Lei, H. He and Y. Ai, *J. Environ. Sci*, 2023, **126**, 470–482.

9 L. Lin, W. Fang, Q. Liang, Y. Xing, M. Sun and H. Luo, *J. Environ. Chem. Eng*, 2024, **12**, 112590.
